# *In vitro* differentiation of fertile sperm from cryopreserved spermatogonia of the endangered endemic cyprinid honmoroko (*Gnathopogon caerulescens*)

**DOI:** 10.1038/srep42852

**Published:** 2017-02-17

**Authors:** Shogo Higaki, Manami Shimada, Kazuaki Kawamoto, Takaaki Todo, Toshihiro Kawasaki, Ikuo Tooyama, Yasuhiro Fujioka, Noriyoshi Sakai, Tatsuyuki Takada

**Affiliations:** 1 Laboratory of Cell Engineering, Department of Pharmaceutical Sciences, Ritsumeikan University, Kusatsu, Shiga 525-8577, Japan; 2 Laboratory of Cell Engineering, Graduate School of Life Sciences, Ritsumeikan University, Kusatsu, Shiga 525-8577, Japan; 3 Genetic Strains Research Center, National Institute of Genetics, Mishima, Shizuoka 411-8540, Japan; 4 Molecular Neuroscience Research Center, Shiga University of Medical Science, Otsu, Shiga, 520-2192, Japan; 5 Lake Biwa Museum, Oroshimo 1091, Kusatsu, Shiga 525-0001, Japan

## Abstract

Many endemic fish species are threatened with extinction. Conservation strategies and the restoration of endemic fish after extinction must therefore be investigated. Although sperm cryopreservation is indispensable for the conservation of endangered fishes, the limited number of mature fish and limited availability (volume and period) of sperm from small endemic fish hinders the optimization and practical use of this material. In this report, we demonstrate the *in vitro* differentiation of fertile sperm from cryopreserved spermatogonia of juveniles of the endangered small cyprinid honmoroko (*Gnathopogon caerulescens*), which is endemic to Lake Biwa in Japan. The entire process of spermatogenesis was recapitulated *in vitro* using cryopreserved spermatogonia of non-spawning adult and juvenile fish. The differentiation of sperm from spermatogonia was captured as a time-lapse video and confirmed by 5-ethynyl-2′-deoxyuridine (EdU) incorporation into sperm. Fertility was demonstrated by artificial insemination. These results suggest that the combination of cryopreservation of spermatogonia and *in vitro* sperm differentiation will provide a new and promising strategy for the preservation of paternal genetic materials.

Animal populations are sustained by a balance among various species, and the loss of biodiversity influences entire ecosystems^1^. Many endemic fishes have been classified as endangered species, and the threats to these species have recently become more immediate^2^. Important technical approaches to restoring these species involve the preservation of sperm, eggs or embryos. Although sperm cryopreservation has been reported in large fish such as salmonids and in experimental model fish such as zebrafish (*Danio rerio*) and medaka (*Oryzias latipes*)^3^, few reports have described sperm cryopreservation in small endangered fish species. Systematic studies of cryopreservation in small endangered fish are prevented by their small volume, the short durations of their spawning periods^4^, inter-individual variation in quality, and the limited number of mature fish available. In addition, eggs, which are required for testing the fertility of cryopreserved sperm, are not available except during the spawning period^5^, and cryopreservation methods for eggs have not been established because of their large size, high sensitivity to chilling, and low membrane permeability^6^,^7^. Although transplantation of germ cells (primordial germ cells^8^, spermatogonia^9^, and oogonia^10^) into surrogates has been reported for producing both eggs and sperm, allogeneic surrogates will not be available after the extinction of an endangered fish, and validation of suitable xenogeneic surrogates takes time. Therefore, it is important to prepare multiple strategies for the conservation of critically endangered fishes.

The use of spermatogenic cells other than sperm is beneficial for the preservation of critically endangered fish species because it expands the possibilities for preservation and allows the use of non-spawning males and even juveniles as genetic resources. Furthermore, the preservation of undifferentiated male germ cells, spermatogonia, provides a unique advantage because, after transplantation into a surrogate, these cells can potentially differentiate into eggs as well as sperm^11^. Therefore, the use of spermatogonia is a practical method for the preservation of both sperm and eggs. *In vitro* production of sperm from spermatogonia has been reported for three fish species, Japanese eel (*Anguilla japonica*)^12^, medaka^13^, and zebrafish^14^,^15^. However, such sperm has only been confirmed to be fertile for zebrafish, using sperm differentiated from freshly prepared spermatogonia^14^ or using spermatogonial stem cells cultured for up to 1 month^15^. No report has described the *in vitro* differentiation of fertile sperm from the cryopreserved spermatogonia of juveniles or non-spawning adult fish. Here, we describe the establishment of culture and cryopreservation conditions for spermatogonia of the critically endangered species *Gnathopogon caerulescens* and demonstrate the differentiation of fertile sperm from cryopreserved spermatogonia using juveniles and non-spawning adults.

## Results

### Histological analysis of spermatogenic cells in *G. caerulescens*

**Figure 1:**
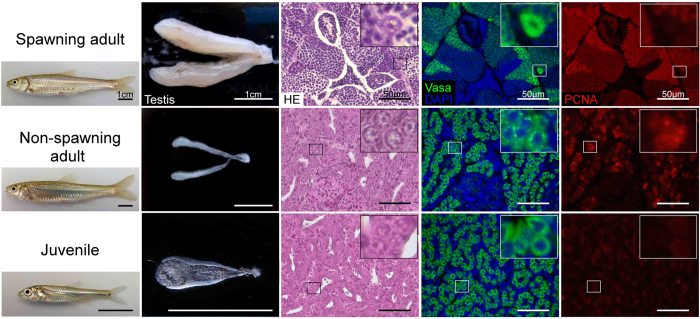
Histological analysis of testes from adult and juvenile *Gnathopogon caerulescens*. Testes were sampled from spawning (May) and non-spawning (September) adults and from juvenile fish. Images show the gross morphology of the fish and testes; adjacent testicular sections are stained with haematoxylin-eosin (HE) and for Vasa (green) and proliferating cell nuclear antigen (PCNA, red). Nuclei were stained with DAPI (blue). The images shown are representative of ten different male *G. caerulescens* per group. The insets in each panel show high-magnification images of spermatogonia. Spawning testes (May) were thick and plump with ivory white coloration, whereas non-spawning adult (September) and juvenile (5-month-old) testes were thin and threadlike with translucent white coloration. Experiment reproduced ten times. Scales are indicated in the figure.

To identify spermatogenic cells such as spermatogonia, spermatocytes, spermatids, and sperm in *G. caerulescens*, testicular sections of matured spawning (May) and non-spawning (September) adults, as well as juvenile fish (approx. 5 months old, ~4 cm in body length), were analysed histologically. Based on the histological identification of spermatogenic cells, flow cytometric analyses were also performed using propidium iodide (PI) staining and expression of the germ cell marker Vasa^16^. Testes from spawning adults contained all developmental stages of spermatogenic cells^17^, including spermatogonia (0.1%), spermatocytes (11.2%), spermatids, and flagellated sperm (82.2%) (Fig. 1 and Supplementary Fig. S1). Intense Vasa signal was observed in the spermatogonial cytoplasm; Vasa signal was also present in primary spermatocytes, but no signal was detected in secondary spermatocytes, spermatids, or spermatozoa. In the non-spawning testis, spermatogonia with strong Vasa signal were predominant in the seminiferous tubules (Fig. 1). Based on flow cytometry, the population of spermatogenic cells was estimated as follows: spermatogonia, 32.4%; spermatocytes, 1.7%; and somatic cells, 59.2% (Supplementary Fig. S1). Spermatids and sperm were not observed. The proliferation of spermatogonia was detected using PCNA staining. In juvenile fish, spermatogonia with single nucleoli and strong Vasa signal were predominant (49.9%), similar to the non-spawning adult testis (Fig. 1 and Supplementary Fig. S1). However, they showed higher levels of heterochromatin and little PCNA signal in the nucleus, unlike the spermatogonia in the non-spawning testis (Fig. 1). No spermatocytes, spermatids or sperm were detected, and somatic cells composed 39.5% of the samples (Supplementary Fig. S1).

### *In vitro* sperm differentiation from spermatogonia

Enzymatically dissociated testicular cells were grown in either adherent or suspension culture using testicular cell culture medium (TCCM)^18^ supplemented with growth factors and hormones under humidified air at 18 °C.

**Figure 2:**
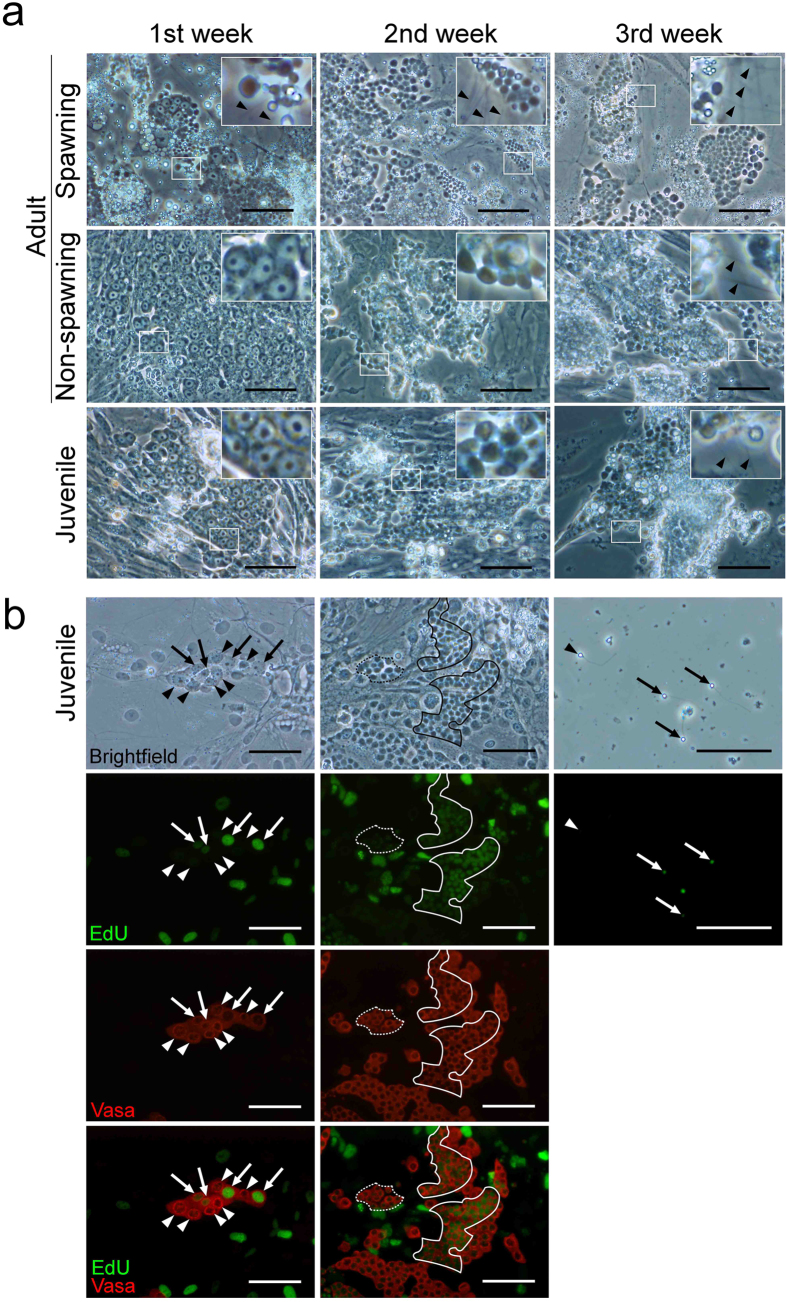
*In vitro* spermatogenesis of *G. caerulescens* in adherent culture. Testicular cells isolated from spawning (May) and non-spawning (September) adult and juvenile testes (5-month-old) were cultured. (**a**) Bright-field images of testicular cell cultures. The insets in each panel represent high-magnification images of the most advanced germ cell types. In the testicular cell cultures from spawning adults, spermatogenic cells of all developmental stages were observed at days 7, 11, and 21. In the testicular cell cultures from non-spawning adults, germ cell colonies of spermatogonia, spermatocytes, and flagellated sperm were observed at days 7, 11, and 18. In testicular cell cultures from juveniles, colonies of spermatogonia, spermatocytes, and flagellated sperm were observed at days 4, 13, and 19. Sperm flagella are indicated by arrowheads. Experiment reproduced six times. Bars represent 100 μm. (**b**) Bright-field images (top) of juvenile testicular cell cultures and fluorescent images for 5-ethynyl-2′-deoxyuridine (EdU, green) and Vasa (red). Nuclei were stained with DAPI (blue). EdU-positive and EdU-negative cells are indicated by the arrows/solid lines and arrowheads/dotted lines, respectively. EdU-positive spermatogonia, spermatocytes, and sperm were observed at days 7, 14, and 21, respectively. Experiment reproduced four times. Bars represent 50 μm.

In the adherent culture of spawning testis, all stages of spermatogenic cells were observed on the fibroblast-like testicular somatic cells during the first week of culture (Fig. 2a). These germ cell colonies underwent spermatogenesis, and motile flagellated sperm were observed at the beginning of the first week in culture. Flagellated sperm were then released into the culture medium. The morphology of these spermatogenic cell colonies *in vitro* was quite similar to that observed in testicular sections (Figs 1 and 2a). Flagellated sperm were produced continuously for approximately 1 month, and the size and number of spermatogenic cell colonies decreased accordingly. Adherent somatic cells proliferated to confluence and detached from the dish, forming a cell sheet that wrapped around the germ cell colonies and terminated sperm production.

In the cultures of non-spawning adult and juvenile testes (Fig. 2), only spermatogonia were observed on the somatic cells during the first week. Then, the spermatogonia differentiated to form spermatocyte colonies during the second week of culture. The differentiation of spermatogonia proceeded via a similar time course in both cultures, and motile, flagellated sperm were observed during the third week (Supplementary movie S1). Thereafter, sperm production decreased gradually as described in the culture of spawning testes. Differentiation of spermatogonia was also monitored using Vasa staining during the culture period (Supplementary Fig. S2).

To confirm the production of sperm from spermatogonia *in vitro,* 5-ethynyl-2′-deoxyuridine (EdU) incorporation was evaluated. EdU-positive spermatogonia, spermatocytes, and flagellated sperm were detected during the first, second, and third weeks of culture when using juvenile testes, respectively (Fig. 2b).

### Cystic spermatogenesis on the surfaces of cell aggregates in suspension culture

**Figure 3:**
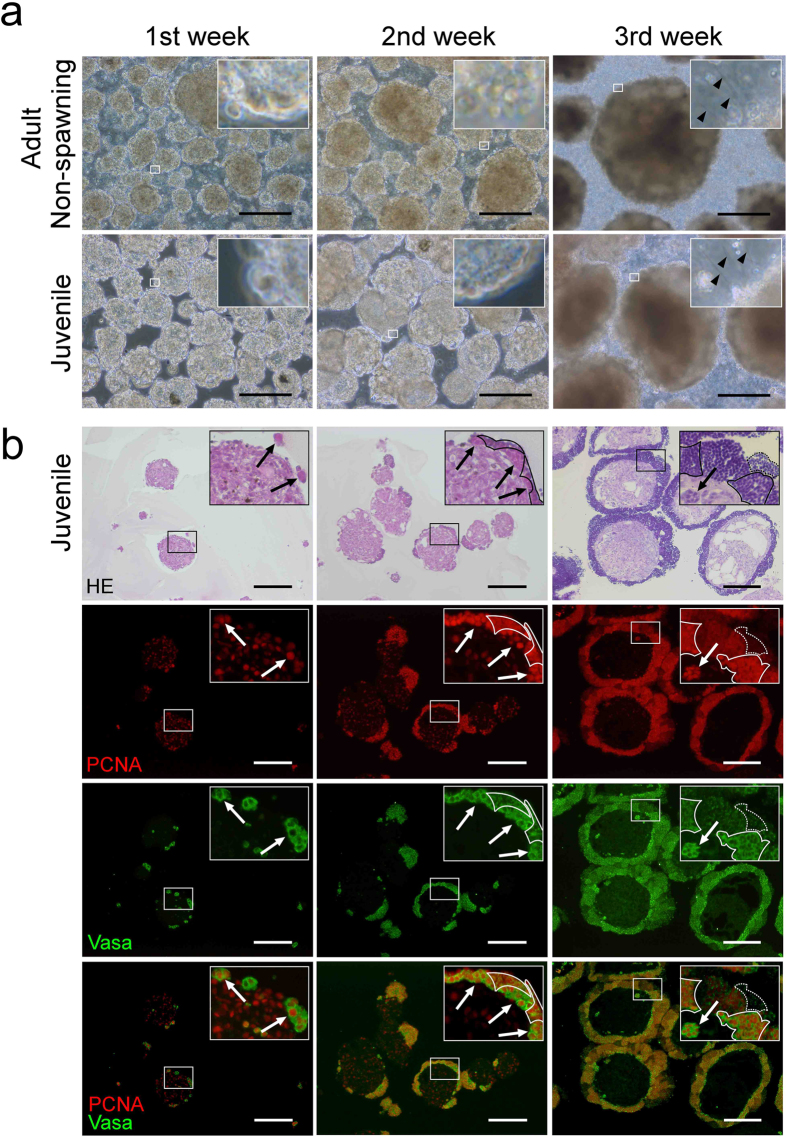
*In vitro* spermatogenesis in suspension culture. Testicular cells isolated from non-spawning adults and juveniles were cultured in suspension. (**a**) Bright-field images of testicular cell cultures. The insets in each panel represent high-magnification images of the selected area. Sperm flagella are indicated by arrowheads. Experiment reproduced four times. Bars represent 200 μm. (**b**) Histological sections of cell aggregates formed in juvenile testicular cell cultures. Adjacent sections were stained with haematoxylin-eosin (HE) (top) and for Vasa (green) and PCNA (red). The insets in each panel represent high-magnification images of the most advanced germ cell types: spermatogonia, spermatocytes, and spermatozoa in the first, second, and third weeks of culture, respectively. Colonies of spermatogonia (arrows), spermatocytes (solid lines), and sperm (dotted lines) are indicated. Experiment reproduced three times. Bars represent 200 μm.

We next tested whether suspension culture could improve spermatogenesis. Dissociated testicular cells autonomously assembled to form spherical aggregates. When non-spawning adult and juvenile testicular cells were cultured, many flagellated sperm appeared on the surfaces of the aggregates during the third week of culture (Fig. 3a and Supplementary movie S2), in a time course similar to that in adherent culture. The flagellated sperm then detached from the aggregates and were released into the culture medium. Cell aggregates grew to 500 μm in diameter in the third week and actively produced sperm for approximately 1 month. Thereafter, the size of the aggregates and the number of flagellated sperm in the culture medium gradually decreased, and the sperm production ceased after approximately 2 months.

Immunohistochemical analysis of the aggregates revealed that a small number of Vasa-positive spermatogonia were scattered on the surfaces of the cell aggregates in the first week, and the spermatogonia then differentiated into spermatocytes, which covered the entire surface. This is the opposite of the cell configuration in the testes, in which Vasa-positive germ cells are located outside the aggregates, with Vasa-negative somatic cells located inside (Fig. 3b). Similar to the testicular germ cells, however, immunostaining for Vasa and PCNA showed the formation of cysts and the synchronous differentiation of spermatogenic cells in the cysts (Fig. 3b).

**Figure 4:**
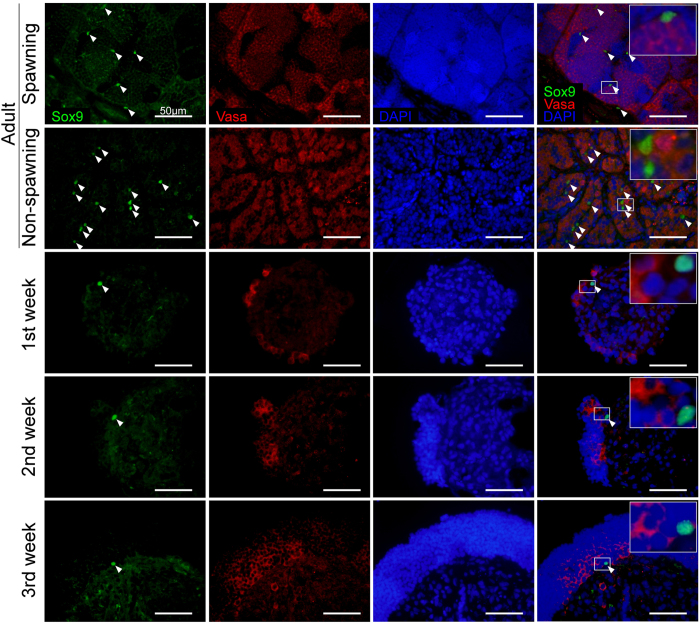
Expression of Sox9 in *in vivo* and *in vitro* spermatogenesis. Testes were sampled from spawning (May) and non-spawning (September) adults. Cell aggregates formed in suspension culture of non-spawning adult testicular cells were sampled at first, second, and third week of culture. Sections were stained for Sox9 (green) and Vasa (red). Nuclei were stained with DAPI (blue). Arrowheads indicate Sox9-positive cells. The insets represent high-magnification images of Sox9-positive cells. Experiment reproduced three times. Bars represent 50 μm.

As Sertoli cells interact directly with germ cells and are prerequisite for spermatogenesis *in vivo*^19^, we investigated the localization of Sertoli cells in *in vitro* culture, by immunostaining of cell aggregate sections with Sox9 antibody^20^,^21^. In spawning testes, Sox9-positive Sertoli cells were frequently found at the periphery of the spermatogonial cysts characterized by the higher level of Vasa-expression and large nuclei, while in non-spawning testes many Sox9-positive Sertoli cells were detected adjacent to the spermatogonia (Fig. 4). In cell aggregates, Sox9-positive cells were observed in close proximity to the spermatogonial cysts throughout the culture period. This result suggests that the spatial arrangement of spematogonia and Sox9-positive Sertoli cells *in vitro* was similar to that of *in vivo*.

### Haploid formation *in vitro*

**Figure 5:**
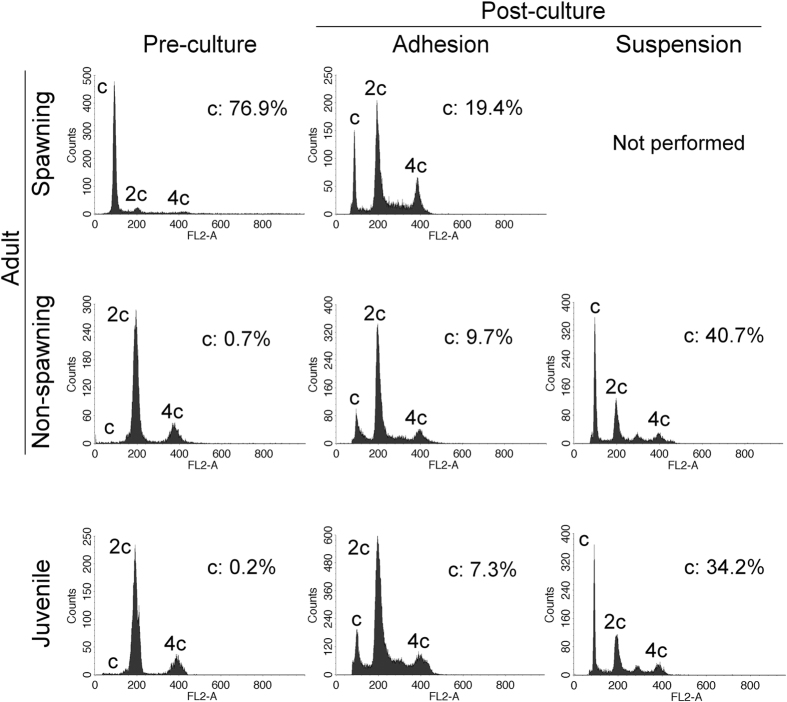
Analysis of the DNA content of testicular cells. The DNA content of the spawning and non-spawning adult and juvenile testes was analysed using flow cytometry before and after culture. Flow cytometric profiles of PI intensity, presented as histograms (FL2-A). Histogram of freshly dissociated testicular cells of spawning and non-spawning adult and juvenile testes (top). Histogram of testicular cells cultured for longer than three weeks in adherent (middle) and suspension (bottom) cultures. The percentages of haploid cells are indicated. The DNA content is indicated by c, 2c, and 4c, representing haploid, diploid, and tetraploid, respectively. Experiment reproduced three times.

To monitor meiosis and haploid formation *in vitro*, the DNA content of pre-culture (freshly dissociated testicular cells) and post-culture cells was analysed by flow cytometry with PI staining (Fig. 5). Haploid cells, sperm and spermatids, were observed only in the spawning testes, not in the non-spawning adult and juvenile testes (Fig. 5 and Supplementary Fig. S1). After the culture, a clear haploid peak was detected in the cultures of non-spawning adult and juvenile testes, as well as those of spawning adults, suggesting that spermatogenic cells underwent successful meiosis *in vitro*. The proportions of haploid cells among cultures from non-spawning adult and juvenile testes were 9.7 and 7.3% in adherent culture and 40.7 and 34.2% in suspension culture, respectively. This suggests that spermatogenesis progressed more efficiently in suspension culture than in adherent culture.

### Efficient sperm production from cryopreserved spermatogonia *in vitro*

The cryopreservation of germ cells is a prerequisite and a key technology for conserving endemic fish. Therefore, the cryopreservation of spermatogonia prepared from non-spawning adults and juvenile testes was examined using rapid (vitrification) cooling method. As spermatogonia are the unipotent stem cells and shares some characteristics with pluripotent stem cells (e.g., self-renewal, high nuclear cytoplasmic ratio, and presence of prominent nucleoli), we used commercially available freezing medium for human iPS cells. After 6-months to 3-years of storage, cell survival rates and differentiation ability of the cryopreserved testicular cells were examined. The survival rates of cryopreserved testicular cells of non-spawning adult and juvenile fish were 64.7 ± 9.1% and 52.2 ± 9.5% (n = 6), respectively. Vitrified cells in either adherent or suspension culture formed many germ cell colonies and differentiated into sperm with motile flagella. The entire process of spermatogenesis was recorded as a time-lapse video (Supplementary movie S3), showing active migration, cell division, and differentiation of spermatogonia of *G. caerulescens*, as in mouse seminiferous tubules^22^. Because uniform somatic cells^23^ are known to proliferate in adherent culture, we attempted to separate the germ cells, which produce less side-scattered light than the somatic cells, using only PI staining and flow cytometry. The germ cells formed 20.9% of the adherent culture (spermatids and sperm: 2.9%, spermatogonia and spermatocytes: 18.0%), with the somatic cells forming 78.3% (Supplementary Fig. S3a). However, in the suspension culture of cryopreserved non-spawning testes, germ cells were predominant, forming 87.6% of the cells (spermatids and sperm: 25.4%, spermatogonia and spermatocytes: 62.2%) (Supplementary Fig. S4b). Accordingly, the proportion of somatic cells in suspension culture was much lower than that in adherent culture (9.9 vs 78.3%, respectively). The suspension culture of vitrified juvenile testes also showed efficient differentiation of sperm (32.0%) and a high proportion of germ cells (75.2%) (Supplementary Fig. S3b). The total number of sperm produced in suspension culture (17.85 ± 1.95 × 10^5^, n = 4) was larger than that of adhesion culture (4.98 ± 1.37 × 10^5^, n = 4) (*P* < 0.05), when cryopreserved non-spawning testicular cells (6.8 × 10^5^ cells) were cultured for 30-days.

### Motility of the sperm differentiated *in vitro*

Initiation of motility of the sperm differentiated *in vitro* was triggered at hypotonic conditions. The sperm in the culture medium were activated immediately by diluting the medium with water more than 10 times (Supplementary movie S4 and S5). The physical parameters of sperm motion were compared to those of *in vivo*-differentiated sperm (Supplementary Fig. S4 and Table S1). The mean values of VCL (curvilinear velocity) and VSL (straight line velocity) in *in vitro*-differentiated sperm (129.6 ± 40.5 μm/s and 106.4 ± 45.8 μm/s, respectively) were both lower than those of *in vivo*-differentiated sperm (269.8 ± 60.3 μm/s and 257.2 ± 69.1 μm/s, respectively) (*P* < 0.05). In addition, linear pattern of movement (LIN = VSL/VCL × 100) of *in vitro*-differentiated sperm (81.7 ± 20.7%) was also lower than that of *in vivo*-sperm (94.8 ± 10.4%) (*P* < 0.05). These results suggest that *in vitro*-sperm have acquired motility at 28 days of culture, but their motility is inferior to that of *in vivo*-sperm regarding the velocity and linearity.

### Fertility of the sperm differentiated *in vitro*

**Figure 6:**
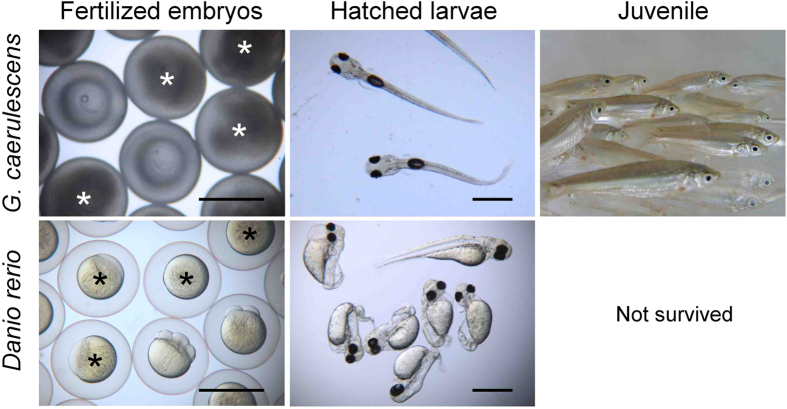
Offspring production using *in vitro* differentiated sperm. Fertilized embryos, hatched larvae, and juveniles developed from *G. caerulescens* (top) and *D. rerio* (bottom) eggs that were artificially inseminated with *in vitro* differentiated sperm from cryopreserved juvenile testicular cells of *G. caerulescens*. The fertilized eggs of *G. caerulescens* reached the somite stage at 24 h post-insemination and hatched at approximately 7 days. The fish grew to approximately 6 cm in body length by 7 months. Fertilized *D. rerio* eggs reached the 4-cell stage at 3 h post-insemination. Asterisks indicate unfertilized eggs. Experiment reproduced nine (*G. caerulescens*) and eight (*D. rerio*) times. Bars represent 1 mm.

To validate the fertility and developmental competence of the sperm differentiated *in vitro*, the sperm were used for *in vitro* fertilization with unfertilized eggs obtained from female *G. caerulescens* by the injection of human chorionic gonadotropin (HCG). As shown in Table 1, *in vitro* differentiated sperm from both fresh and cryopreserved testicular cells were able to fertilize eggs, and most of the fertilized embryos hatched. Within the sperm differentiated from cryopreserved cells, an interaction between the effects of culture methods (adhesion and suspension cultures) and origin of testicular cells (non-spawning adult and juvenile fish) on the fertilization and hatching rates was observed (*P* < 0.05). Suspension culture of cryopreserved spermatogonia yielded higher rates of fertilization and hatching (24.7 and 21.7% in non-spawning adults and 4.8 and 4.1% in juveniles, respectively) than did adherent culture (both values are 0.7% in non spawning adults and 0.6% in juveniles, *P* < 0.05), regardless of the origin of testicular cells. The fertilization and hatching rates of non-spawning adult were both higher than those of juvenile in suspension culture (*P* < 0.05), but not in adherent culture. No fertilized embryos were obtained using sperm subjected to repeated freeze-thaw cycles or to more than 27 days in culture, suggesting that fertilized embryos were not parthenogenotes activated by unknown factor(s) in the culture medium, nor were they the result of contamination with sperm from the spawning male. Offspring were obtained from cryopreserved spermatogonia of juveniles and from non-spawning testes, and these offspring developed normally (Fig. 6). Obvious anomalies, such as the abnormally small eyes^24^ that are a known characteristic of artificially induced gynogenetic haploid *G. caerulescens*, were not observed. In addition, yolk sac absorption and feeding behaviour appeared to be normal.

Because *G. caerulescens* eggs are only available during the spawning period, it is impossible to examine fertility during most of the year, even with cultures of freshly prepared spermatogonia from non-spawning adult and juvenile testes. To overcome this problem, we investigated whether zebrafish eggs, which also belong to the family Cyprinidae and have the same chromosomal number (2n = 50; zebrafish^25^, *G. caerulescens*^26^), can be used to validate the fertility of *G. caerulescens* sperm. Both *in vivo* differentiated sperm collected from spawning *G. caerulescens* and *in vitro* differentiated sperm from non-spawning adults were able to fertilize zebrafish eggs at high rates: 79.1 and 61.4%, respectively. The fertilized eggs also developed and hatched (Fig. 6 and Supplementary Table S2), but the hatching rates were lower than that with allogeneic eggs (35.2 and 11.4% vs 76.5%). *In vitro* differentiated sperm from cryopreserved spermatogonia of non-spawning adults and juveniles were also able to fertilize zebrafish eggs (at 0.3 and 3.5%, respectively). Although the hatched larvae did not display a short and stocky body^27^, which is the most common feature of gynogenetic haploid zebrafish, many of them showed a truncated body axis with severe anomalies in the circulatory system, and they did not survive longer than 4 days.

To confirm resultant larvae are true hybrid of *G. caerulescens* and zebrafish, we performed genotyping and ploidy analysis using genomic PCR and flow cytometry. Amplification of C-terminal region of *Sox9b* ortholog gave a unique band in *G. caerulescens* (328 bp) and zebrafish (248 bp), and both bands were detected in hybrid larvae (Supplementary Fig. S5). In addition, flow cytometry revealed that the DNA content of resultant larvae cells was different from that of the diploid zebrafish embryo and zebrafish sperm. It showed a good agreement with the sum of the DNA of *G. caerulescens* and zebrafish sperm (Supplementary Fig. S6). These results clearly indicate that the resultant larvae were truly hybrids of *G. caerulescens* and zebrafish, not parthenogenotes nor gynogenetic haploid.

## Discussion

This study demonstrates that in the critically endangered endemic cyprinid *G. caerulescens*^28^, spermatogonia from juvenile fish and from non-spawning adults can be cryopreserved by vitrification, and fertile sperm can be differentiated *in vitro*. To the best of our knowledge, this is the first report of *in vitro* differentiation of fertile sperm from fresh and cryopreserved spermatogonia from juvenile and non-spawning adult fish. Furthermore, we reconstituted three-dimensional cystic spermatogenesis *in vitro* using suspension culture, which is more suitable for efficient sperm production than is the adherent culture reported for zebrafish^14^. Of interest, spermatogenesis occurred only on the surface of cell aggregates in suspension culture. This can be explained by the availability of factors, which are required for spermatogenesis such as nutrients, hormones, and growth factors. These factors were provided through the medium in *in vitro* culture, and therefore spermatogenesis could take place in the region with close proximity to these factors. In addition, similarity of the cystic localization of Sox9-positive Sertoli cells and spermatogonia in spawning testes and cell aggregates suggests that *in vitro* spermatogenesis proceeded in a similar manner to that of *in vivo* with regard to the interaction with Sertoli cells, and the presence of Sox9-expressing Sertori cell in the cysts would be necessary for spermatogenesis. This result was consistent with the *Sox9b* expression reported in medaka^29^, in which *Sox9b* is more strongly expressed near spermatogonia compared to more developmentally advanced germ cells. Therefore, the reason that Sox9 signal was not detected in the cysts of later stages of development might be attributed to the lower level of Sox9-expression to detect with this antibody.

The use of spermatogonia permits the use of non-spawning fish, including juveniles, which could not otherwise be used for sperm collection, and it avoids sacrificing spawning males for sperm preservation. In addition, the successful cryopreservation of spermatogonia can be confirmed simply in culture, without the fertilization of eggs from a spawning female. The production of fertile sperm from juvenile testes is particularly advantageous for the conservation of critically endangered fish, given that the cold winters faced by *G. caerulescens* cause the loss of most juveniles before sexual maturity^30^.

Here, we have demonstrated efficient *in vitro* spermatogenesis, which requires only 3 weeks from spermatogonia to flagellated sperm, even in juveniles. Spermatogenic cells observed in juvenile testes are mitotically inactive spermatogonia. Therefore, this culture condition represents an entire stage of meiosis. *G. caerulescens* in the wild requires approximately one year to reach sexual maturity^31^, and the differentiation of sperm from spermatogonia requires approximately 6 months *in vivo* (from September to March)^32^. Despite such rapid spermatogenesis *in vitro,* differentiated sperm were fertile and showed no detrimental effects on further development, suggesting that this culture can recapitulate normal meiosis *in vitro*. Shortening the sperm production using *in vitro* spermatogenesis from juvenile fish will be an advantage for the propagation of endangered species.

High fertility and hatching rates (24.7 and 21.7%, respectively) were achieved using suspension cultures with high haploid rates (25.4%) from tissues of non-spawning adults (Supplementary Fig. S3b). The low rates of fertilization and hatching (less than 1%) using adherent culture of vitrified cells may be attributable to the small number of sperm used for *in vitro* fertilization (Supplementary Fig. S3a). Sperm differentiation appears to be favored by the formation of three-dimensional cysts on the surfaces of the cell aggregates and the limited proliferation of somatic cells in the suspension culture. Similar reciprocal proliferation of germ cells and somatic cells has also been reported in testicular cell culture for the rainbow trout *Oncorhynchus mykiss*^33^. These high rates of fertilization and hatching in the suspension culture of cryopreserved spermatogonia were similar to those reported in zebrafish using sperm differentiated from adult testes *in vitro* (15% fertilization rate)^34^ and spermatocytes of medaka (30% hatching rate)^35^. Further, these rates were comparable to those of cryopreserved sperm in zebrafish (33% fertilization rate)^36^, goldfish (23% fertilization rate)^37^, loach (29% hatching rate)^38^, and shoveljaw carp (22% hatching rate)^39^, suggesting that this *in vitro* differentiation strategy using spermatogonia is valid. On the other hand, motility of the sperm is a major importance for fertilization and we confirmed that *in vitro*-produced sperm already acquired motility by activation. Although how these sperm acquired motility is not clear, we noticed that the floating sperm, which detached from the cysts, appeared to have acquired motility but not the sperm still attached to the cysts. Detailed investigation of the releasing process from the cysts might provide some information of the motility acquisition. The lower velocity and linearity of *in vitro*-produced sperm compared to the sperm produced *in vivo,* possibly declined the fertility. Further improvement will be required in culture system to differentiate sperm, which have comparable motility to the sperm produced *in vivo* (Supplementary movie S4, S5, Fig. S4, and Table S1).

Although DNA replication was detected in germ cells under this culture condition, the self-renewal of spermatogonia did not appear to occur dominantly: the number of spermatogonia and differentiated sperm gradually decreased with prolonged cell culture, as reported in zebrafish^14^. Therefore, establishment of the culture, which allows the self-renewal of undifferentiated spermatogonia or spermatogonial stem cells^15^, would be important for the propagation of endangered endemic fish because it allows for an unlimited supply of spermatogonia and resultant sperm. Because this *in vitro* spermatogenesis is simple and takes less time and effort than *in vivo* sperm production, it might be applicable for other endangered fish species.

We also demonstrated that zebrafish eggs can be used to validate the fertility of *G. caerulescens* sperm differentiated *in vitro. G. caerulescens* eggs are highly valuable because they are only available during the spawning season; further, it is difficult to control ovulation, and the eggs show quality variation between individuals. Zebrafish eggs, by contrast, are readily available throughout the year, and their homogeneous genetic background provides more consistent results. In addition, the transparency of zebrafish eggs allows the scoring of fertility within 2 h after fertilization, whereas *G. caerulescens* eggs are opaque and require 24 h (until the somite stage) to confirm fertilization.

Because fish are the most diverse group of vertebrates and show vast differences in reproduction, conservation strategies must be established using the fish of interest. In this study, we demonstrated that the combination of cryopreservation of spermatogonia and *in vitro* differentiation of sperm using non-spawning adults and juveniles of *G. caerulescens* provides not only a new strategy for the conservation of endangered endemic fish but also a simple *in vitro* model of vertebrate spermatogenesis. Further studies, which may allow self-renewal of spermatogonia and the production of mature eggs *in vitro* or by the xenogeneic transplantation of undifferentiated germ cells, will be the next step, and these key technologies will make it possible to accomplish the complete regeneration of endangered endemic fish.

## Methods

### Fish

Adult male honmoroko (*G. caerulescens*) (1 to 2 years old) were reared in outdoor ponds near Lake Biwa and were collected during the spawning period (May, spring) and during the non-spawning period (September, late summer; January, winter). Juvenile *G. caerulescens* males (approx. 5 months old, ~4 cm in body length) were grown from embryos and kept in indoor tanks in the laboratory under a natural light-dark cycle at 24 ± 2 °C.

To prepare unfertilized eggs for artificial insemination assays, adult female *G. caerulescens* (>1 year old) were collected on the day before use during the spawning period. The wild-type zebrafish strain RIKEN WT was obtained from the RIKEN Brain Science Institute (Saitama, Japan) and maintained in the laboratory under a 12 h light:12 h dark photoperiod at 27 ± 1 °C. All experiments were approved by the committee on laboratory animal care and use at Ritsumeikan University (Shiga, Japan), and performed according to the guidelines of Ritsumeikan University.

### Preparation of testes

For the testicular cell culture experiments, male fish were euthanized by immersion in ethyl p-aminobenzoate (250 μg/ml; Benzocaine, Sigma-Aldrich, St. Louis, MO, USA) (for adult fish) or ice-cold water (for juvenile fish) and their surfaces were disinfected with 70% (v/v) ethanol. The testes were aseptically dissected out from the fishes and transferred into Ca^2+^/Mg^2+^-free phosphate-buffered saline (PBS) containing 0.5% (v/v) commercial bleach (Heiter, Kao, Tokyo, Japan) for sterilization. After 2-min immersion, the testes were washed three times in sterile PBS for 2 min each. All procedures were performed at room temperature unless otherwise specified.

### Histological analysis of testes

Testicular fragments of randomly selected ten individual fish per group were fixed with 4% (w/v) paraformaldehyde in PBS supplemented with 10% (v/v) acetic acid at 4 °C overnight. The fixed tissues were washed with PBS, dehydrated in ethanol and xylene, embedded in paraffin, and cut into 5-μm-thick sections.

After deparaffinization, some of the sections were stained with haematoxylin and eosin (HE). Adjacent sections were immunohistochemically stained. Briefly, deparaffinized sections were incubated in 0.05% (v/v) citraconic anhydride solution (pH 7.4; Immunosaver, Nissin EM, Tokyo, Japan) for 45 min at 98 °C for antigen retrieval. Following the permeabilization with 0.2% (v/v) Triton X-100 for 10 min, the sections were blocked in PBS containing 4% (v/v) normal goat serum for 30 min. Thereafter, tissue sections were incubated overnight at 4 °C with diluted antibodies in PBS containing 4% normal goat serum. The following primary antibodies were used: rabbit anti-zebrafish Vasa (GTX128306: GeneTex, Irvine, CA, USA; diluted 1:1000), rat anti-*Drosophila* Vasa (Developmental Studies Hybridoma Bank [DSHB], Iowa City, IA, USA; diluted 1:200), mouse anti-rat PCNA (P8825: Sigma-Aldrich, St. Louis, MO, USA; diluted 1:2000), and rabbit anti-human Sox9 (AB5535: Millipore, Temecula, CA, USA; diluted 1:500). Next, they were incubated with the appropriate secondary antibodies diluted in PBS for 45 min. The following secondary antibodies were used: Alexa Fluor 488-conjugated goat anti-rabbit IgG (A-11008: Invitrogen, Carlsbad, CA, USA; diluted 1:500), Alexa Fluor 568-conjugated goat anti-mouse IgG (A-11004: Invitrogen; diluted 1:500), and Alexa Fluor 568-conjugated goat anti-rat IgG (ab175476: Abcam, Cambridge, MA, USA; diluted 1:100). Nuclei were counterstained with 4′,6′-diamidino-2-phenylindole (DAPI, Dojindo Laboratory) for 20 min. Negative control samples received identical staining preparations, except the primary antibody. Images were obtained at ×400 magnification using an inverted fluorescence microscope (IX70: Olympus, Tokyo, Japan) equipped with a CCD camera (VB-7000, Keyence, Osaka, Japan). Developmental stages of spermatogenetic cells were identified according to the classification of closely related cyprinid zebrafish^40^.

### Culture of testicular cells

Primary cell culture was performed as described for male germ cell cultures of zebrafish^18^ with several modifications. Freshly dissected testicular fragments were minced and dissociated in Leibovitz’s L-15 medium (L5520: Sigma-Aldrich) containing 500 U/ml collagenase type IV (17104-019: Life Technologies, Gaithersburg, MD, USA) for 2 h at 28 °C under constant shaking (140 rpm) with gentle pipetting every 20 min. The cell suspension was diluted ten times with 4-(2-hydroxyethyl)-1-piperazineethanesulfonic acid (HEPES) buffered Leibovitz’s L-15 medium containing 1% (w/v) bovine serum albumin fraction V (BSA, Sigma-Aldrich) and filtered through a 100-μm pore-size nylon mesh (Cell strainer, Corning, Corning, NY, USA). After centrifugation at 120 × g for 7 min, the cell pellet was resuspended in testicular cell culture medium (TCCM)^18^ supplemented with 10% (v/v) foetal bovine serum (FBS, Invitrogen), 3% (v/v) common carp (*Cyprinus carpio*) serum, 100 ng/ml epidermal growth factor (EGF, Peprotech, Rocky Hill, NJ, USA), 100 ng/ml basic fibroblast growth factor (bFGF, Peprotech), 100 ng/ml insulin-like growth factor-I (IGF-I, Peprotech), 10 μM forskolin (Sigma-Aldrich), 0.1 mM β-mercaptoethanol (Sigma-Aldrich), 10 IU/ml human chorionic gonadotropin (HCG, ASKA Pharmaceutical, Tokyo, Japan), 10 IU/ml pregnant mare’s serum gonadotropin (PMSG, ASKA Pharmaceutical), 50 ng/ml 11-ketotestosterone (11-KT, Cosmo Bio, Tokyo, Japan), 100 ng/ml 17β-estradiol (E2, Wako Pure Chemicals, Osaka, Japan), and 10 ng/ml 17α,20β-dihydroxy-4-pregnen-3-one (DHP, Toronto Research Chemicals, North York, NO, Canada). The cells originated from one testis of spawning adult were then seeded in three 35-mm plastic dishes containing 1.5 ml of the medium. In the case of non-spawning adult and juvenile fish, the cells isolated from one testis and five paired testes (in total ten testes) were seeded in one dish, respectively. The cells were then incubated at 18 °C in humidified air, and the medium was changed every 3 or 4 days. For adherent culture, 0.1% (w/v) gelatine-coated tissue culture dishes (3000-035: AGC Techno Glass, Shizuoka, Japan) were used. Cell culture images under adhesion and suspension conditions were obtained at ×200 and ×100 magnification, respectively, using an inverted microscope.

The composition of the TCCM was Leibovitz’s L-15 medium supplemented with 2 mM L-glutamine (Sigma-Aldrich), 50 U/ml penicillin–50 μg/ml streptomycin (Nacalai Tesque, Kyoto, Japan), 100 μg/ml Kanamycin sulfate (Nacalai Tesque), 800 μM CaCl_2_ (Wako Pure Chemicals), 200 μg/ml L-arginine (Sigma-Aldrich), 20 μg/ml L-aspartic acid (Sigma-Aldrich), 15 μg/ml L-histidine–HCl (Sigma-Aldrich), 72.5 μg/ml L-lysine–HCl (Sigma-Aldrich), 20 μg/ml L-proline (ICN Biomed, Milano, Italy), 0.5% BSA, 10 mM HEPES (Nacalai Tesque), 2 embryos per ml of common carp embryo extract, and 20% Milli-Q water (Millipore)^18^. Common carp embryo extract was prepared according to the procedure described previously^41^. Briefly, 2-day-old larvae obtained through artificial insemination^42^ were sterilized with Ringer’s solution for fresh water fish (116 mM NaCl, 2.9 mM KCl, 1.8 mM CaCl_2_, and 5 mM HEPES in water) containing 0.5% commercial bleach and washed twice with the Ringer’s solution. The washed embryos were then suspended in the same volume of Leibovitz’s L-15 medium and homogenized twenty times on ice with a Dounce tissue grinder. The resulting homogenate was diluted with Leibovitz’s L-15 medium to 200 embryos/ml, centrifuged at 6500 × g for 10 min at 4 °C, and the supernatant was collected. The supernatant was sterilized through a 0.2-μm pore filter and stored at −80 °C.

### Immunochemical staining of cultured cells

Adherent cells were washed twice with PBS and fixed with 4% paraformaldehyde in PBS for 15 min. Following two washes with PBS, the cells were permeabilized by 0.2% Triton X-100 for 10 min, blocked with 4% normal goat serum, and stained with rabbit anti-Vasa antibody and Cy3-conjugated goat anti-rabbit IgG (AP132C: Millipore; diluted 1:500) or Alexa Fluor 488-conjugated goat anti-rabbit IgG. For the suspension culture, cell aggregates were harvested by centrifugation at 10 × g for 1 min and embedded in iPGell (GenoStaff, Tokyo, Japan), followed by fixation in 4% paraformaldehyde at 4 °C overnight. The fixed cell aggregates were then embedded in paraffin, cut into 5-μm-thick sections, and stained with antibodies against Vasa, PCNA, and Sox9 as described. Nuclei were counterstained with DAPI. Fluorescence images of cultured cells under adhesion and suspension conditions were obtained at ×400 and ×40 magnification using an inverted fluorescence microscope.

### DNA replication of spermatogenic cells *in vitro*

To trace the progress of spermatogenesis *in vitro*, the Click-iT EdU cell proliferation assay kit (C10337, Invitrogen) was used for labelling and the detection of DNA replication, according to the manufacturer’s instructions. In short, EdU was added to the culture medium at a final concentration of 10 μM on the second day of adherent testicular cell culture prepared from juvenile fish. After 48 h of culture, the medium was replaced by fresh medium without EdU. At the time of detecting the DNA replication, adherent cells were washed twice with PBS and fixed with 4% PFA on the culture dish. Following two washes with 3% BSA in PBS, the cells were permeabilized by 0.5% Triton X-100 for 20 min and EdU-labeled DNA was conjugated to Alexa Fluor 488-N_3_ by CuSO_4_-catalyzed click chemistry^43^. For suspended cells including sperm, cells were collected by centrifugation of the culture medium at 800 × g for 5 min. The cells in the pellet was fixed, permeabilized, stained for EdU in the centrifugation tubes, and the stained cells were spread onto hydrophilic glass slides. Fluorescent images were obtained at ×400 magnification using an inverted microscope.

### Flow cytometric analyses

For the flow cytometric analyses of cell composition in testes, dissociated testicular cells were washed by centrifugation at 800 × g for 7 min with PBS and fixed with 70% (v/v) ice-cold ethanol for 30 min. After centrifugation, the pelleted cells were washed by centrifugation with PBS containing 0.1% BSA and 0.1% saponin. The cells were then re-suspended in 4% normal goat serum for blocking. After 30 min incubation, rabbit anti-Vasa antibody was added (diluted 1:100) and incubated one hour at room temperature. Following two washes by centrifugation with PBS containing BSA and saponin, Alexa Fluor 488-conjugated goat anti-rabbit IgG was added (diluted 1:500) and incubated for 45 min. The cells were then washed by centrifugation with PBS containing BSA and saponin and treated with 1 mg/ml RNase A (Nacalai Tesque) for 30 min at 37 °C. After centrifugation, the cell nuclei were stained with 50 μg/ml of PI (Wako Pure Chemicals) for 15 min in the dark. These testicular cells were filtered through a 40-μm nylon mesh (N-N0330T: NBC Meshtec, Tokyo, Japan), diluted with PBS containing 1% BSA, and subjected to flow cytometry.

For the analyses of cells grown in adherent culture, germ cells and somatic cells were prepared separately. Briefly, the germ cell colonies were detached from the adherent somatic cells by using PBS for 20 min at room temperature. The detached germ cells and the supernatant medium containing sperm were combined to prepare the germ cell fraction, which was centrifuged at 800 × g for 7 min and washed twice with PBS. The pelleted cells were dissociated using 0.06% (w/v) trypsin (27250-042: Gibco, Grand Island, NY, USA) containing 1.32 mM ethylene diamine tetraacetic acid for 10 min. The remaining somatic cells were harvested by trypsinization to obtain the somatic cell fraction. The trypsin was neutralized by adding Leibovitz’s L-15 medium containing 10% FBS and the cells in the suspension was washed again by centrifugation with PBS. These cells were fixed and the nuclei were stained with PI as described above. These germ or somatic cell fractions were filtered through a 40-μm nylon mesh, and subjected to flow cytometry. For the cultured cells in suspension, the whole culture medium containing cell aggregates and detached sperm was centrifuged at 800 × g for 7 min, and the pelleted cells were treated as described for the germ cell above. For pre-culture controls, dissociated cells from adult and juvenile testes were prepared and analysed as described in testicular cells.

To determine the ploidy of hybrid embryos produced by *G. caerulescens* sperm and zebrafish eggs described below, hatched hybrid and zebrafish larvae were dissociated in Leibovitz’s L-15 medium containing 500 U/ml collagenase for 1 h at 28 °C with gentle pipetting every 10 min. The dissociated cells were washed twice by centrifugation at 120 × g for 5 min with PBS, and the pelleted cells were fixed and stained with PI as described. Squeezed sperm from mature male *G. caerulescens* and zebrafish was also fixed, stained, and analysed as haploid controls.

Flow cytometry was performed on a FACSCalibur (BD Biosciences, San Jose, CA, USA) equipped with an argon laser (488-nm). The fluorescence signals of the Vasa (FL1-H, 530/30 nm bandpass) and PI (FL2-A, 585/42 bandpass) were collected in the logarithmic and linear mode, respectively. Forward scatter (FSC-H) and side scatter (SSC-H) signals were collected using linear and logarithmic mode, respectively. The spectral overlap between Alexa Fluor 488 and PI was corrected by hardware compensation. To identify spermatogenic cells of various developmental stage and somatic cells in testes, cells were separated using SSC and FL1. In the case of ploidy analysis only PI staining was performed and analysed using SSC and FL2. A minimum of 3,000 events were collected for each sample with “LO” (12 μl/min) flow rate. Data were analysed with CellQuest Pro software (ver. 6.0, BD Biosciences).

### Cryopreservation of testicular cells

To preserve testicular cells, dissociated testicular cells were cryopreserved using rapid (vitrification) cooling method. Briefly, the dissociated testicular cells were washed with ten volumes of HEPES-buffered Leibovitz’s L-15 medium containing 1% BSA. Aliquots containing dissociated cells of two pairs of testes (for non-spawning adult fish) or five pairs of testes (for juvenile fish) were separated and then centrifuged at 120 × g for 7 min. The pelleted cells were re-suspended in 200 μl of DAP213 solution (2 M DMSO, 1 M acetamide, and 3 M propylene glycol) (RCHEFM001: ReproCELL, Kanagawa, Japan)^44^, transferred into CryoTube Vial (377267: Thermo Scientific, Waltham, MA, USA), and cooled rapidly by plunging it into liquid nitrogen within 15-sec. For warming, the cryopreserved vial was removed from the liquid nitrogen tank and the cells were warmed quickly by adding the 1 ml pre-warmed (37 °C) Leibovitz’s L-15 medium supplemented with 10% FBS (thawing solution) within 15-sec. The cell suspension was diluted with 9 ml thawing solution and washed by centrifugation at 120 × g for 3 min. Following the removal of the supernatant, the cell pellet was re-suspended with the cell culture medium and cultured as described above.

Viabilities of cryopreserved testicular cells were assessed on the bases of the intactness of the plasma membrane using the trypan blue exclusion test just after the dilution with the thawing solution. In short, 10 μl of 0.5% (w/v) trypan blue solution (Kanto Chemical, Tokyo, Japan) was added to a same volume of the diluted testicular cell suspension and incubated for 3 min. The number of trypan blue-negative and -positive cells were counted using a hematocytometer, and the percentages of membrane intact cells were calculated according to the following formula: percentages of membrane intact cells = number of trypan blue-negative cells/number of total cells.

### Time-lapse imaging of cultured cells

Cryopreserved testicular cells of non-spawning adult were counted using a hematocytometer soon after thawing and seeded in a gelatin-coated tissue culture dish (35-mm) containing 4.5 ml of the medium at a very low cell density (approx. 1,000 cells per dish). The culture medium was sealed with 2 ml of mineral oil (M8410, Sigma-Aldrich) to prevent evaporation. The cell culture dish was then placed on the stage of an inverted microscope (PrimoVert HDcam, Carl Zeiss, Oberkochen, Germany) mounted in an incubator (MB-6110C, Mitsuboshi Boeki, Hyogo, Japan) set to 18 °C. Images were captured once every 100 sec at ×100 magnification and played back at 5 frames per sec (fps). The time-lapse video generated using Labscope software (ver. 2.0, Carl Zeiss) on an iPad (Apple, Cupertino, CA, USA) was then converted to mp4 format at 40 fps using every third frame, giving a final playback speed of 12,000 × real speed; video conversion was performed using AviUtl (ver. 1.00, http://spring-fragrance.mints.ne.jp/aviutl/).

### Analysis of sperm motility

Sperm motility was analysed according to the previous study^45^ with slight modifications. Briefly, culture medium containing sperm differentiated from non-spawning adult testes was centrifuged at 800 × g for 7 min to concentrate the sperm. The majority of the supernatant was discarded, and the cell pellet was resuspended in the remaining medium (<100 μl). A 10-μl of concentrated sperm suspension was placed on a self-made glass chamber (approximately 150-μm deep), cover slipped, and then the sperm were activated by adding a 100 μl deionized water. Images were recorded at ×100 magnification over 2 min from 5 sec before the sperm activation using an inverted microscope. Since motility of *G. caerulescens* sperm decreases very rapidly after mixing with water, sperm motility was analysed using the first 10 sec movie from the water addition. In each sample, the swimming path of 50 individual motile sperm was manually tracked using ImageJ (ver. 1.51 h; National Institutes of Health, Bethesda, MD, USA) plugin MTrackJ (ver. 1.5.1; Erasmus University, Rotterdam, Netherlands)^46^. Each track was followed for 1 sec divided into seven steps. The kinematic parameters that define sperm motility, including curvilinear velocity (VCL, the actual velocity along the trajectory), straight line velocity (VSL, the straight line distance between the start and end points of the track divided by the time of the track), and linearity (LIN, the ratio of net distance moved to total path distance (VSL/VCL × 100))^47^, were measured. Freshly squeezed sperm from mature male *G. caerulescens* was also analysed after 2 × 10^4^ times dilution with culture medium.

### Fertilization assay

Unfertilized eggs were collected from mature female *G. caerulescens* and zebrafish as reported^48^,^49^. A batch of eggs collected from one female was fertilized with the *in vitro*-differentiated sperm recovered from one 35-mm dish. Number of sperm was counted using hemocytometer after the sperm collection. The sperm suspension was concentrated by the centrifugation at 800 × g for 7 min, added to the unfertilized eggs in the dish, and the dish was swirled for 1 min to mix the sperm and eggs well. Then, dechlorinated tap water was added gradually with shaking, and the eggs were incubated at 23 °C for *G. caerulescens* eggs or at 28 °C for zebrafish eggs. We checked the embryonic development at the somite stage (for *G. caerulescens* eggs, approximately 24 h post-insemination) or the four-cell stage (for zebrafish eggs, approximately 3 h post-insemination) and at the hatching stage (for *G. caerulescens* and zebrafish eggs, 7 and 3 days post-insemination, respectively). Somite stage was chosen for checking the fertilization of *G. caerulescens* eggs, because the cleavage could not be seen through their opaque chorion. At somite stage, however, the fertilized eggs became distinguishable from unfertilized eggs as unfertilized eggs ruptured in the chorion and darkened. While in zebrafish egg, four-cell stage was chosen for checking the fertilization, because unfertilized zebrafish eggs have occasionally undergone a single, asymmetric cleavage. Freshly squeezed sperm from mature male *G. caerulescens* was used as a positive control. Squeezed sperm cultured for 27 days in the same culture conditions as the testicular cells was used as a negative control. Both control sperm samples were used at the sperm-egg ratio of the sperm recovered from one male per batch of eggs from one female.

### Genotyping

Genomic DNA was prepared from hatched larvae of *G. caerulescence*, hybrid produced by *G. caerulescence* sperm and zebrafish egg, and zebrafish using PureLink Genomic DNA Mini Kit (Thermo Fisher Scientific, Waltham, USA). C-terminal region of *Sox9b* orthologs were amplified using primers 5′-cagatcaagacggagcagctgag-3′ and 5′-gggtctggacagctgtgtgtagac-3′, which locate in exon 3 of zebrafish sequence.

The PCR reactions were performed using *Ex Taq* DNA polymerase (Takara Bio, Shiga, Japan) according to the manufacturer’s instructions with 10 ng of genomic DNA as a template. Amplification program was initial denaturation at 98 °C for 1 min, and 35 cycles of denaturation at 98 °C for 10 sec, annealing at 60 °C for 30 sec, and extension at 72 °C for 30 sec, followed by a final extension at 72 °C for 5 min. The PCR products were separated on 3% (w/v) agarose gel electrophoresis.

### Statistical analyses

Prior to statistical analyses, the percentage data (LIN for sperm motility and fertilization and hatching rates for fertilization assays) were transformed into arcsine values through arcsine square root transformation. Differences between the values of sperm kinematic parameters (VCL, VSL, and LIN) of *in vitro* and *in vivo* differentiated sperm were analysed using Student’s T-test. The numbers of differentiated sperm from cryopreserved non-spawning adult fish under adhesion and suspension conditions were also compared by Student’s T-test. In the sperm produced from cryopreserved cells, the effects of culture methods (adhesion vs. suspension) and origin of testicular cells (non-spawning adult vs. juvenile fish) on the fertilization and hatching rates were analysed by two-way analysis of variance (ANOVA) followed by Sidak’s *post hoc* test. A computer program (SPSS for Windows, Version 12.0, SPSS Inc., IL, USA) was used for statistical analyses.

## Additional Information

**How to cite this article**: Higaki, S. *et al*. *In vitro* differentiation of fertile sperm from cryopreserved spermatogonia of the endangered endemic cyprinid honmoroko (*Gnathopogon caerulescens*). *Sci. Rep.* **7**, 42852; doi: 10.1038/srep42852 (2017).

**Publisher's note:** Springer Nature remains neutral with regard to jurisdictional claims in published maps and institutional affiliations.

## Supplementary Material

Supplementary Information

Supplementary movie S1

Supplementary movie S2

Supplementary movie S3

Supplementary movie S4

Supplementary movie S5

## Figures and Tables

**Table 1:** Fertility of *in vitro* differentiated sperm.

Testicular cell condition	Origin of testicular cells	Culture condition	No. of eggs (No. of replicates)	Somite stage embryos (%)	Hatching rate (%)
Fresh	Spawning adult	Squeezed sperm	6012 (n = 10)	82.0 ± 24.9	76.5 ± 27.2
Fresh	Spawning adult	Squeezed sperm cultured for 27-day	1670 (n = 5)	0	0
Fresh	Spawning adult	Adhesion Frozen-killed	4789 (n = 5)	0	0
—	Water	Not cultured	965 (n = 2)	0	0
Fresh	Spawning adult	Adhesion	1698 (n = 4)	5.8 ± 5.6	2.7 ± 3.0
Cryopreserved	Non-spawning adult	Adhesion	2575 (n = 9)	0.7 ± 1.0	0.7 ± 1.0
Cryopreserved	Juvenile	Adhesion	2894 (n = 9)	0.6 ± 1.4	0.6 ± 1.4
Cryopreserved	Non-spawning adult	Suspension	3891 (n = 9)	24.7 ± 14.9^*^	21.7 ± 12.8^*^
Cryopreserved	Juvenile	Suspension	2464 (n = 9)	4.8 ± 2.3^*#^	4.1 ± 1.8^*#^

Adherent and suspension cultures were performed for at least 27 days. Data shown are the sum of independent experiments (n = 2–10). The values are expressed as the means ± standard deviation (%). The average number of egg used for one experiment was 434.8 ± 241.7. ^*^Values (mean ± standard deviation of nine independent experiments) with asterisks indicate significant differences from the corresponding values obtained for cryopreserved testicular cells under adhesion culture (*P* < 0.05). ^#^Values (mean ± standard deviation of nine independent experiments) with hash marks indicate significant differences from the values obtained for cryopreserved testicular cells of non-spawning adult fish under suspension culture (*P* < 0.05).
