# Supplementary material for: In vitro differentiation of fertile sperm from cryopreserved spermatogonia of the endangered endemic cyprinid honmoroko (Gnathopogon caerulescens)
Source: Sci Rep. 2017 Feb 17;7:42852. doi: 10.1038/srep42852 (PMC5314417; doi:10.1038/srep42852)
Supplement: Supplementary Information [file srep42852-s1.pdf]

Supplementary Information for

In vitro differentiation of fertile sperm from cryopreserved spermatogonia of the endangered endemic cyprinid honmoroko (_Gnathopogon caerulescens)_

Shogo Higaki, Manami Shimada, Kazuaki Kawamoto, Takaaki Todo, Toshihiro Kawasaki, Ikuo Tooyama, Yasuhiro Fujioka, Noriyoshi Sakai, Tatsuyuki Takada

Correspondence to: Tatsuyuki Takada, ttakada@ph.ritsumei.ac.jp

This PDF file includes

Figures & Figure legends S1 to S6

Supplementary Table S1 to S2

Movie legends S1 to S5

Supplementary Figure & Figure legends

Supplementary Figure S1.

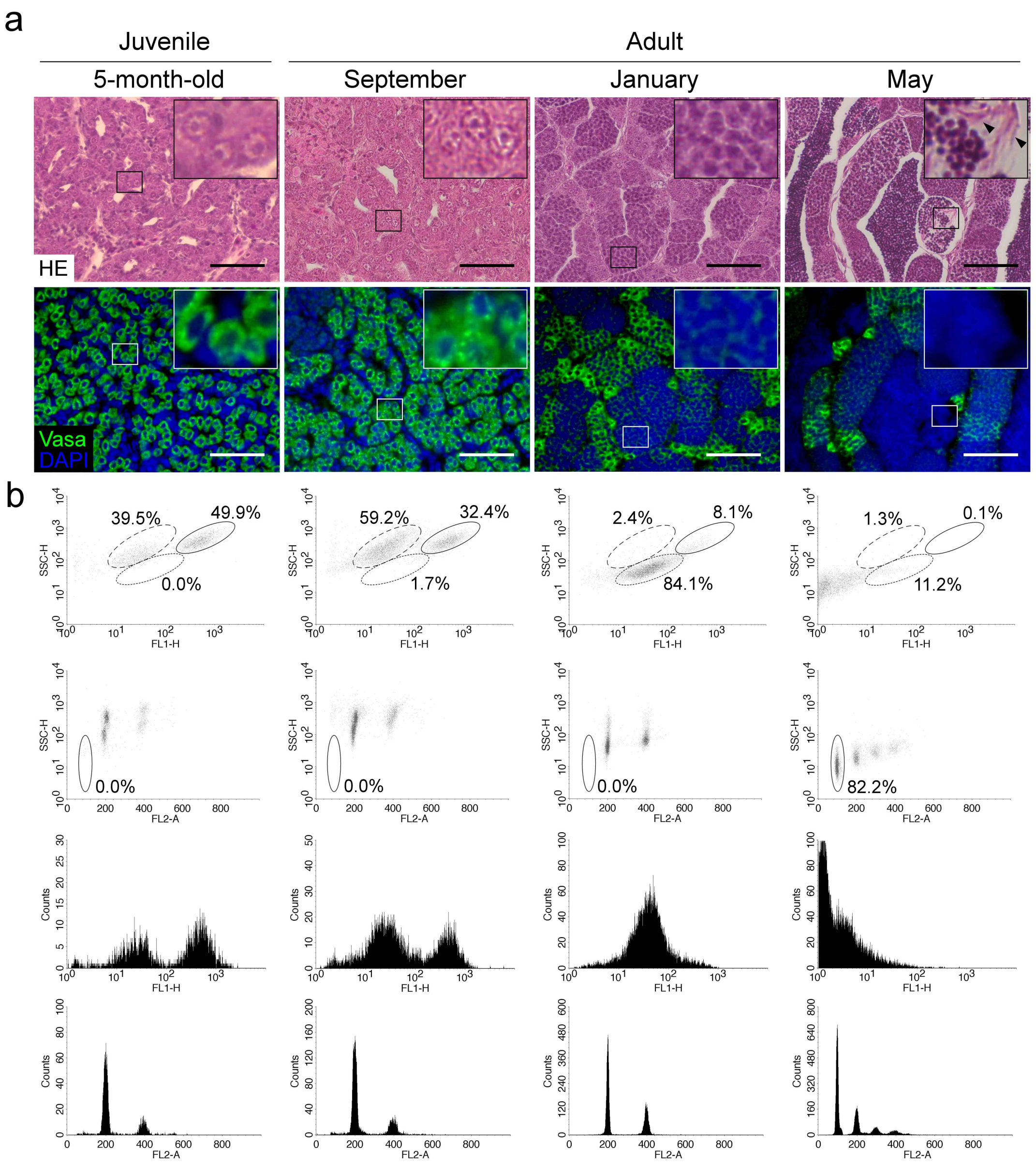

Supplementary Fig. S1. Histological and flow cytometric analyses of testicular cells of *Gnathopogon caerulescens*. Testes of juvenile (5 months old) and adult fish in the non-spawning period (September and January) and spawning period (May) were analysed.

(a) Adjacent sections of the testes were stained with haematoxylin-eosin (HE) and immunostained for Vasa protein (green). Nuclei were stained with DAPI (blue). The insets in each panel represent high-magnification images of the selected area containing

the most advanced germ cell types: spermatogonia in juveniles and adults in September, primary spermatocytes in January, and spermatozoa in May in adults. Only spermatogonia were observed in both the juvenile and adult testes in September. Differentiation of spermatocytes and sperm was confirmed in adult testes in January and May, respectively. The images shown are representative of ten different male *G. caerulescens* from each group. Bars represent 50 μm. (b) Flow cytometric profiles of testicular cells were obtained using Vasa and PI intensities (FL1-H and FL2-H, respectively) and side-scattered light (SSC-H). Spermatogonia, spermatocytes, and testicular somatic cells are represented by solid, dotted, and dashed ellipses, respectively, in the dot plots of SSC-H/FL1-H. Haploid cells (spermatids and spermatozoa) are enclosed by solid ellipses in the dot plots of SSC-H/FL2-A. The percentages of the gated cells are indicated next to the gate. Experiment reproduced at least four times.

Supplementary Figure S2.

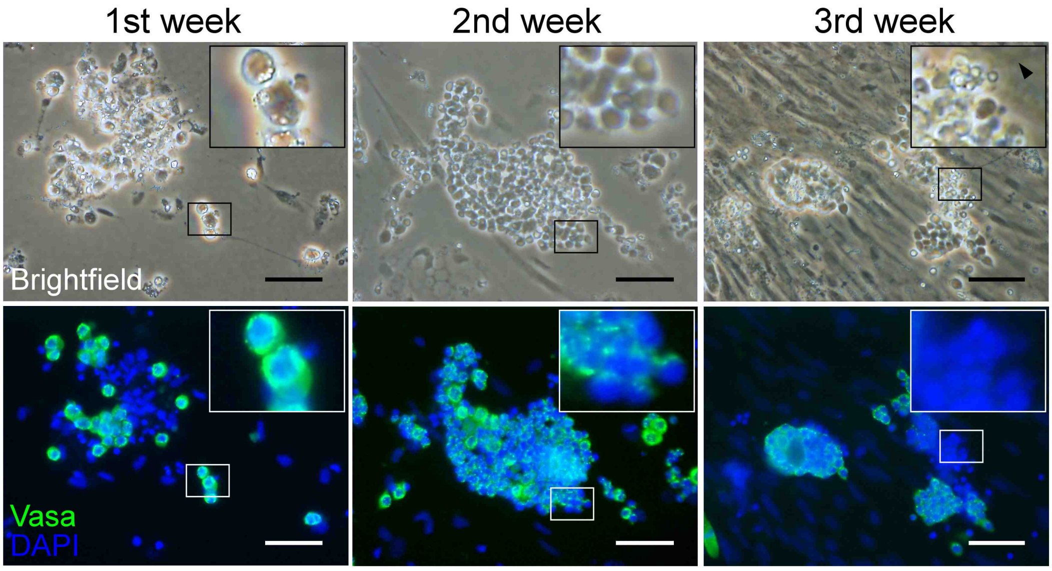

Supplementary Fig. S2. Immunostaining of Vasa in adherent culture of juvenile testes.

Bright-field images of testicular cell cultures at the time points indicated in the figure (top). Immunostaining of the testicular cells with Vasa (green) (bottom). Nuclei were stained with DAPI (blue). The insets in each panel represent high-magnification images of the selected area showing the most advanced germ cell types: spermatogonia at day 7, spermatocytes at day 14, and sperm at day 21. Sperm flagella are indicated by arrowheads. Experiment reproduced four times. Bars represent 50 μm.

Supplementary Figure S3.

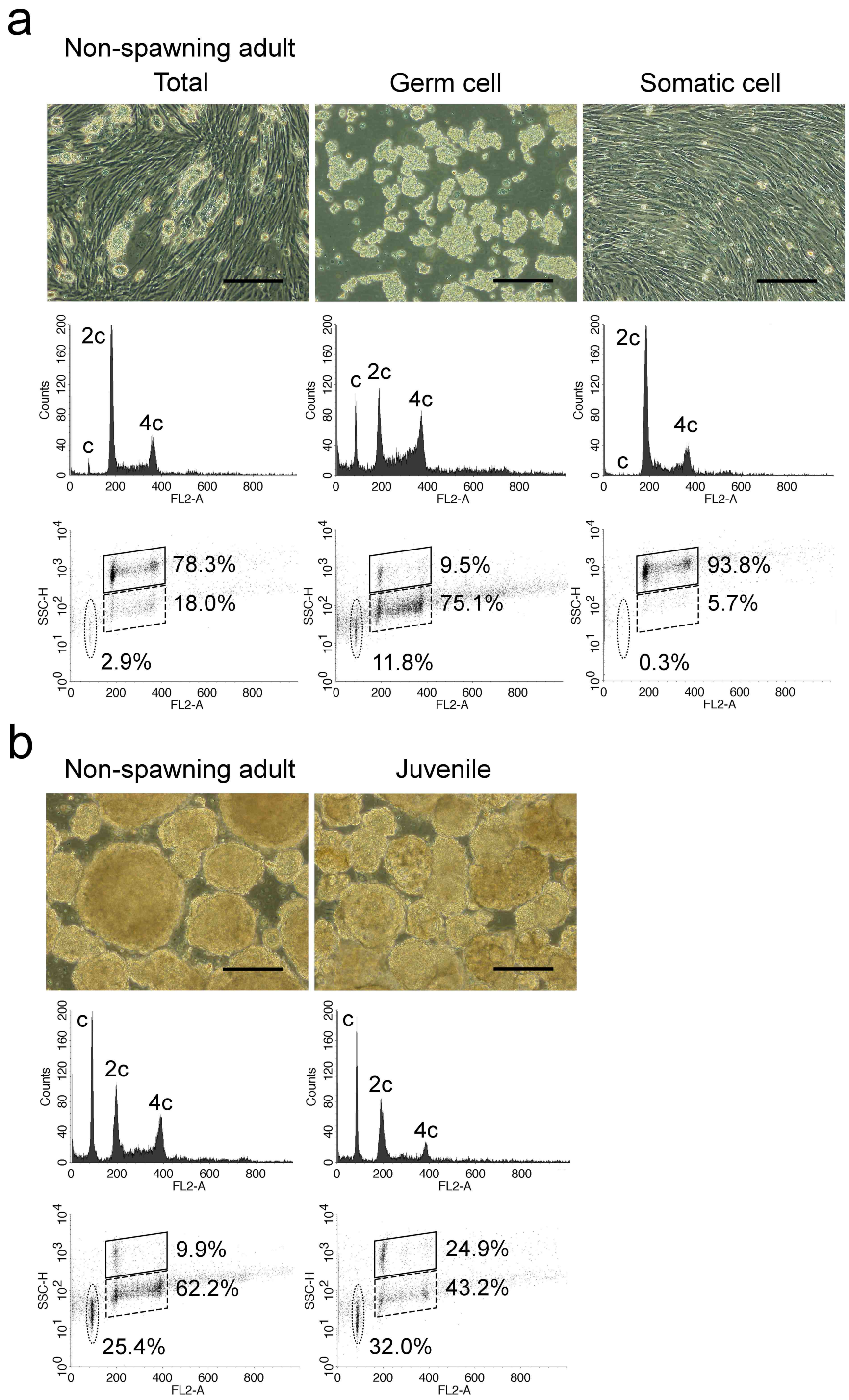

Supplementary Fig. S3. Identification of germ and somatic cells in the culture using flow cytometry.

(a) Germ cell and somatic cell fractions prepared from adherent culture of cryopreserved non-spawning (September) adult testes at day 26. The germ cells and

adherent somatic cell fractions were recovered separately and analysed independently after reconstitution. Experiment reproduced seven times. Bars represent 200 μm. (b) Flow cytometric profile plotted by PI intensity (FL2-A) and side-scattered light (SSC-H). The cell populations enclosed by solid, dashed, and dotted lines contain testicular somatic cells, diploid and tetraploid germ cells (spermatogonia and spermatocytes), and haploid cells (spermatids and spermatozoa), respectively. The percentages next to the outlined areas indicate the percentages of gated cells out of the total cell population. The DNA content is indicated as c, 2c, and 4c, which represent haploid, diploid, and tetraploid, respectively. Experiment reproduced three times.

Supplementary Figure S4.

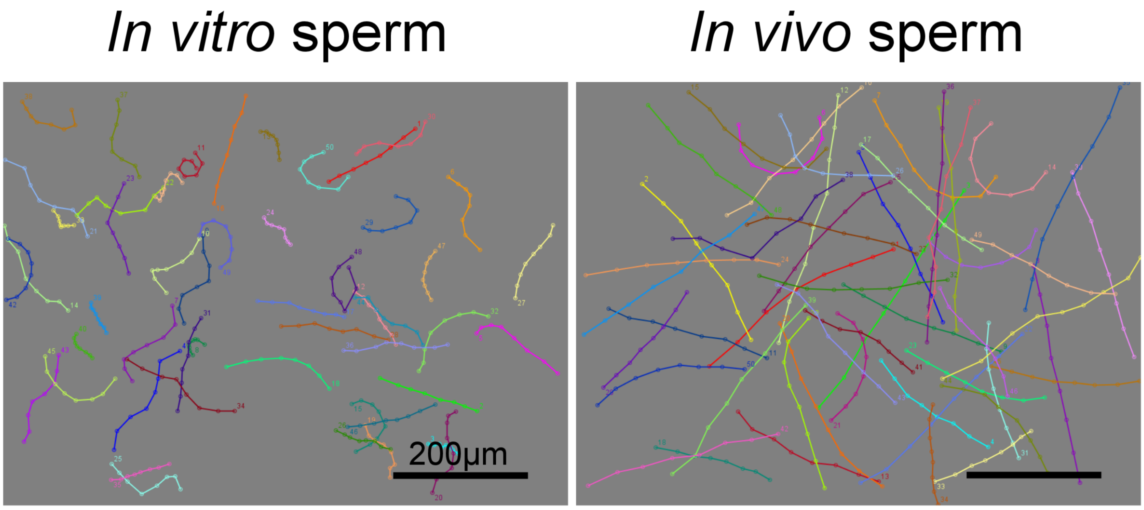

Supplementary Fig. S4. Tracks of activated sperm differentiated *in vitro* and *in vivo*.

In vitro differentiated sperm was obtained from suspension testicular cell cultures of non-spawning (October) adult fish at day 28. While *in vivo* differentiated sperm was used soon after the sperm collection. Fifty tracks were generated by the accumulation of successive eight video frames during 1 sec. Experiment reproduced five times. Bars represent 200 µm.

Supplementary Figure S5. Genotyping of hybrids.

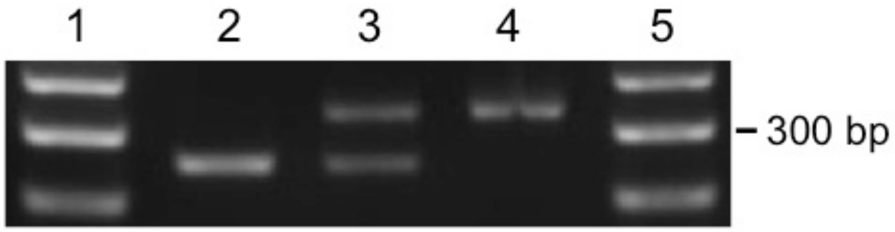

Supplementary Fig. S5. Genotyping of hybrids.

Genomic DNA prepared from hatched larva of *G. caerulescence*, hybrid produced by *G. caerulescence* sperm and zebrafish egg, and zebrafish were PCR amplified with *Sox9b* specific primers and the products were separated on 3% agarose gel electrophoresis. Lanes 1 and 5: 100 bp ladder marker. Lane2: zebrafish, Lane3: hybrid, Lane 4: *G. caerulescens*

Supplementary Figure S6.

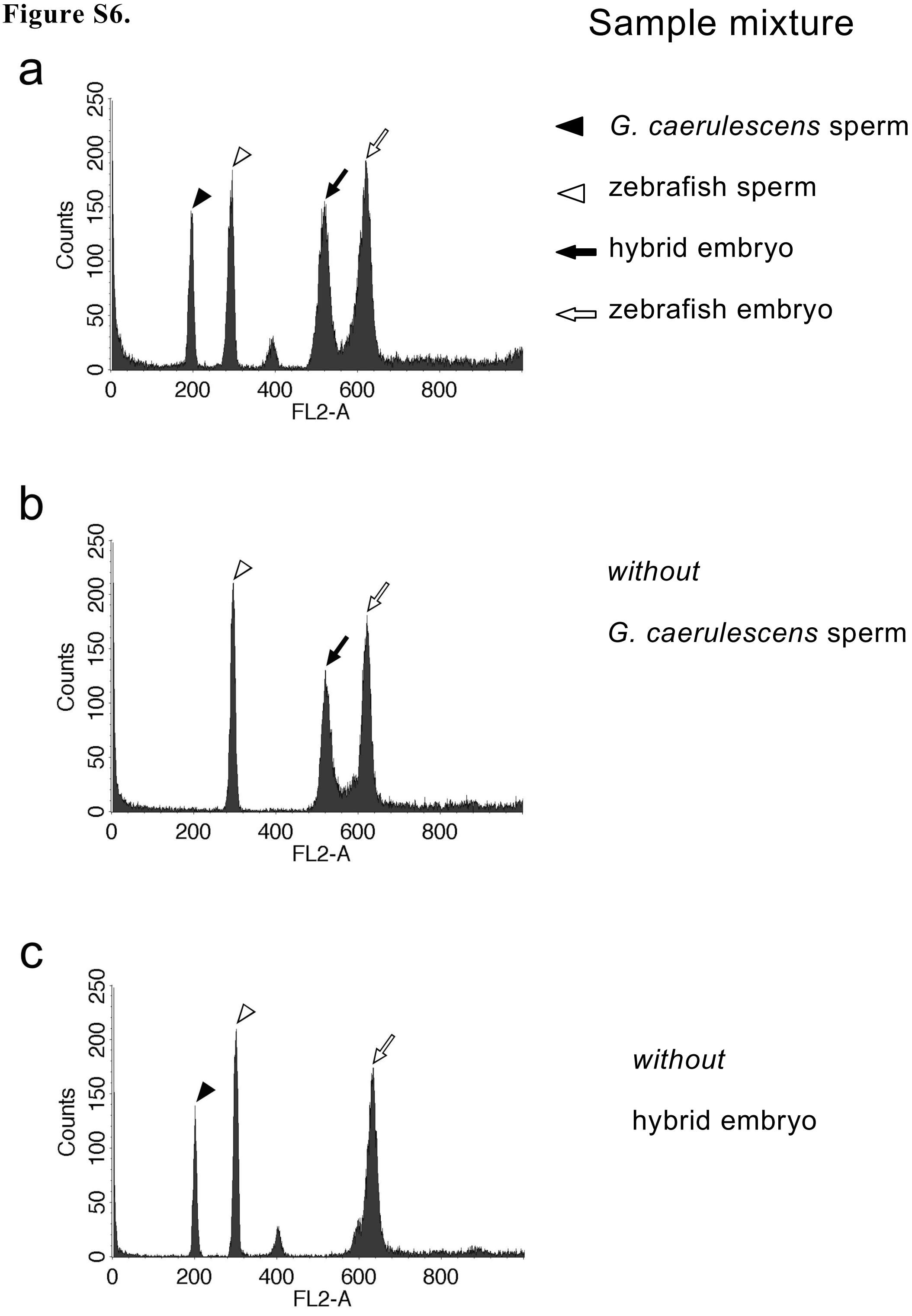

Supplementary Fig. S6. DNA content analysis of hybrid embryos.

The ploidy of hybrid embryos made by *G. caerulescens* sperm and zebrafish eggs was analysed using flow cytometry. Flow cytometric profiles of PI intensity (FL2-A) were

presented as histograms. To validate relative DNA content among cells of different origin (*G. caerulescens* sperm, zebrafish sperm, hybrid embryo, and zebrafish embryo) precisely, samples were mixed prior to the PI staining and analyzed as a mixture (a). To identify the peak of *G. caerulescens* sperm and hybrid embryo, corresponding sample was eliminated from the mixture of all samples (b, c). (a) Histogram of the cell mixture of *G. caerulescens* sperm, zebrafish sperm, hybrid embryo, and zebrafish embryo. (b) Histogram of the cell mixture of zebrafish sperm, hybrid embryo, and zebrafish embryo (without *G. caerulescens* sperm) (c) Histogram of the cell mixture of *G. caerulescens* sperm, zebrafish sperm, and zebrafish embryo (without hybrid embryo). Filled and open arrowheads indicate the peaks of sperm of *G. caerulescens* and zebrafish, respectively. Filled and open arrows indicate the peaks of hybrid embryos produced by *G. caerulescens* sperm and zebrafish eggs, and zebrafish embryo, respectively. Experiment reproduced three times.

Supplementary Table S1. Motility of the sperm differentiated *in vitro* and *in vivo*.

Parameter	*in vitro* sperm	*in vivo* sperm
VCL (μm/s)	129.6 ± 40.5*	269.8 ± 60.3
VSL (μm/s)	106.4 ± 45.8*	257.2 ± 69.1
LIN (%)	81.7 ± 20.7*	94.8 ± 10.4

VCL (curvilinear velocity) and VSL (straight line velocity) were calculated by tracking the trajectory of the individual sperm differentiated *in vitro* and *in vivo* (Supplementary Figure S6). Linear pattern of movement (LIN = VSL/VCL × 100) was also calculated. *Values (mean ± standard deviation of fifty independent sperm) with asterisks indicate significant differences from the corresponding values of *in vivo*-differentiated sperm ($P < 0.05$).

Supplementary Table S2. Fertility of *in vitro* differentiated sperm using zebrafish (*Danio rerio*) eggs

Origin of testicular cells	Testicular cell condition	Culture condition	No. of eggs (No. of replicates)	4-cell stage embryos (%)	Hatched embryos (%)
Spawning adult	Fresh	Squeezed sperm	1561 (n=6)	79.1±11.1	35.2±7.6
Spawning adult	Fresh	Adherent Frozen-killed	637 (n=4)	0	0
Water	No sperm	Not cultured	536 (n=4)	0	0
Spawning adult	Fresh	Adherent	1709 (n=8)	38.3±27.3	19.6±12.7
Non-spawning adult	Fresh	Adherent	2538 (n=7)	61.4±18.9	11.4±8.4
Juvenile	Fresh	Adherent	1672 (n=6)	3.7±2.5	1.7±1.9
Non-spawning adult	Cryopreserved	Adherent	2120 (n=8)	0.3±0.5	0.3±0.5
Juvenile	Cryopreserved	Adherent	1567 (n=8)	3.5±2.4	1.4±1.2

Values were means ± standard deviation of indicated independent experiments. The average number of egg used for one experiment was 242.0±158.0.

Supplementary movie legends

Supplementary movie S1. Motile sperm differentiated *in vitro* in adherent culture.

Testicular cells freshly isolated from a non-spawning (September) adult were cultured. The movie was recorded at day 21. The bar represents 50 μm.

Supplementary movie S2. Motile sperm differentiated *in vitro* in suspension culture.

Testicular cells freshly isolated from a non-spawning (September) adult were cultured. The movie was recorded at day 19. The bar represents 50 μm.

Supplementary movie S3. *In vitro* spermatogenesis of *G. caerulescens*.

Cryopreserved testicular cells isolated from a non-spawning (September) adult were cultured in adherent culture. The time-lapse movie shows the period from 4 to 18 days after cell plating. The bar represents 100 μm.

Supplementary movie S4. Activation of *in vitro* differentiated sperm.

Testicular cells freshly isolated from a non-spawning (October) adult were cultured under suspension condition. The movie was recorded at day 28. Elapsed time from the addition of water is presented. The bar represents 200 μm.

Supplementary movie S5. Activation of *in vivo* differentiated sperm.

Sperm was squeezed from an artificially matured adult fish. The movie was recorded soon after the sperm collection. Elapsed time from the addition of water is presented. The bar represents 200 μm.
